# Additive effects of alkali metals on Cu-modified CH_3_NH_3_PbI_3−*δ*_Cl_*δ*_ photovoltaic devices†

**DOI:** 10.1039/c9ra03068a

**Published:** 2019-08-05

**Authors:** Naoki Ueoka, Takeo Oku, Atsushi Suzuki

**Affiliations:** Department of Materials Science, The University of Shiga Prefecture 2500 Hassaka Hikone Shiga 522-8533 Japan oku@mat.usp.ac.jp

## Abstract

We investigated the addition of alkali metal elements (namely Na^+^, K^+^, Rb^+^, and Cs^+^) to Cu-modified CH_3_NH_3_PbI_3−*δ*_Cl_*δ*_ photovoltaic devices and their effects on the photovoltaic properties and electronic structure. The open-circuit voltage was increased by CuBr_2_ addition to the CH_3_NH_3_PbI_3−*δ*_Cl_*δ*_ precursor solution. The series resistance was decreased by simultaneous addition of CuBr_2_ and RbI, which increased the external quantum efficiencies in the range of 300–500 nm, and the short-circuit current density. The energy gap of the perovskite crystal increased through CuBr_2_ addition, which we also confirmed by first-principles calculations. Charge carrier generation was observed in the range of 300–500 nm as an increase of the external quantum efficiency, owing to the partial density of states contributed by alkali metal elements. Calculations suggested that the Gibbs energies were decreased by incorporation of alkali metal elements into the perovskite crystals. The conversion efficiency was maintained for 7 weeks for devices with added CuBr_2_ and RbI.

## Abstract 2

Addition of alkali metal elements (Na^+^, K^+^, Rb^+^, and Cs^+^) to Cu-modified CH_3_NH_3_PbI_3−*δ*_Cl*_δ_* devices improved the photovoltaic properties.

## Graphical abstract

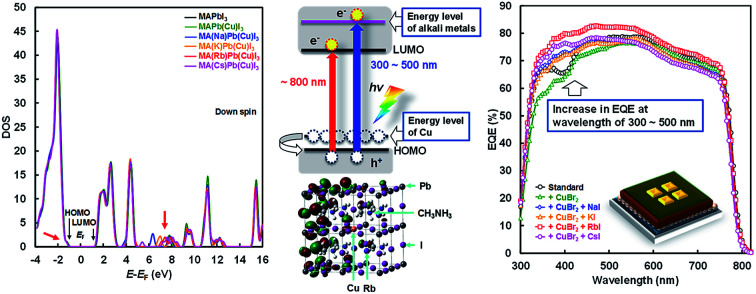

## Introduction

Studies of methylammonium lead halide perovskite solar cells started in 2009, when a conversion efficiency of 3.9% was reported.^1^ Some devices have since yielded conversion efficiencies of more than 20% as studies have expanded globally,^2–5^ with expectations for these perovskite solar cells to be used as next-generation solar cells.^6–9^ Easy fabrication by spin-coating enables rapid development, and mass production of advanced perovskite solar cells will enable their the practical use.^10–14^ TiO_2_ layers have been often used for the perovskite solar cells, and they have an important role as electron transport and hole blocking layers.^15–17^

In general, the perovskite structure has a formula of ABX_3_, where A = CH_3_NH_3_^+^ (MA^+^), B = Pb^2+^, and X = I^−^, which is formed by mixing MAI and PbI_2_. Since 2014, CH_3_NH_3_PbI_3−*δ*_Cl_*δ*_-type compounds have been widely studied,^18–22^ and the introduction of Cl^−^ is beneficial both to perovskite formation and to improve photovoltaic properties.^23–27^ These perovskite compounds can be formed by reacting a mixture of the precursors CH_3_NH_3_I and PbCl_2_ in a 3 : 1 molar ratio based on the following reactions:^28^

1 PbCl_2_ + 3CH_3_NH_3_I → PbI_2_ + CH_3_NH_3_I + 2CH_3_NH_3_Cl (∼20 °C)

2 PbI_2_ + CH_3_NH_3_I + 2CH_3_NH_3_Cl → CH_3_NH_3_PbI_3_ + 2CH_3_NH_3_Cl (g)↑ (140 °C)

3 PbI_2_ + *x*CH_3_NH_3_I + *y*CH_3_NH_3_Cl → (CH_3_NH_3_)_*x*+*y*_PbI_2+*x*_Cl_*y*_ → CH_3_NH_3_PbI_3_ + CH_3_NH_3_Cl (g)↑ (140 °C)

Owing to the formation of intermediates and solvent evaporation, perovskite grains are gradually formed by annealing. Iodine has been substituted by Cl^−^ in this reaction, with the expectation of improved carrier diffusion.^29^ In 2016, Chang *et al.* reported on the growth mechanism of perovskite grains through grazing-incidence wide-angle X-ray scattering measurements, which confirmed that annealing conditions of 140 °C for 12 min were appropriate.^30^

High conversion efficiencies are required for practical applications of perovskite solar cells. Elemental doping is one method to achieve a high conversion efficiency. Doped perovskite structures have been obtained by adding various compounds to the perovskite precursor solutions, and various dopants to the perovskite crystals, such as CH(NH_2_)_2_^+^ (FA^+^), Cs^+^, and Rb^+^ at MA^+^ sites, Sn^2+^ at Pb^2+^ sites, and Br^−^ and Cl^−^ at I^−^ sites.^31–35^ Although photovoltaic properties have been improved by tuning of the electronic structure and surface morphologies, excessive doping decreases conversion efficiencies. Therefore, it is important to control the atomic arrangement and composition of the crystals.

Perovskite solar cells are also required to be stable. Even if cells have high conversion efficiencies, low stability will prevent their practical application. CH_3_NH_3_PbI_3_ perovskite grains decompose and transform to PbI_2_ in the presence of light, oxygen, high temperature and humidity, through the following reaction:^36^

4 4CH_3_NH_3_PbI_3_ + O_2_ → 4PbI_2_ + 2I_2_ + 2H_2_O + 4CH_3_NH_2_

It has been reported that CH(NH_2_)_2_PbI_3_ (FAPbI_3_) is more thermally stable than MAPbI_3_, and the stability has been confirmed to improve through introduction of Br^−^ and Cl^−^, based on first-principles calculations.^37–39^ Because the 5p orbital of the I atom makes the main contribution to the conduction band of perovskite crystal, a blue shift of the band gap occurs through introduction of halides, which becomes more pronounced in the order: Cl^−^ > Br^−^ > I^−^.^40^ Furthermore, Sn has been used to replace Pb, forming Pb-free perovskite crystals, which have a wider range of light absorption owing to a decrease in the band gap. Such devices offer increased current densities and lower toxicity.

As recently reported, Pb–Sn–Cu ternary perovskite solar cells exhibiting multiple crystal orientations have improved charge transport properties.^41^ However, fabrication in a glove box under an inert gas is required because of the easy oxidation of Sn^2+^ to Sn^4+^ at room temperature. In addition, the ionic radius of Cu^2+^ is considerably smaller than that of Sn^2+^. It has been suggested that stable perovskite crystals might be formed by Sn/Cu substitution at Pb sites, as shown in calculations of the tolerance factors.^42^ However, there have been few reports on Cu doped perovskite solar cells, and the small ionic radius of Cu might influence the steric stability of perovskite structures. It has been reported that a perovskite structure with added Co^2+^ changes from cubic to tetragonal,^43^ and control of the crystal structure is important.

In our previous work, photovoltaic properties of perovskite solar cells were improved by adding CuX (X = I, Br, or Cl) to the perovskite precursors through the use of an air-blowing method.^44–47^ Cells with added CuBr featured larger perovskite grains and improved conversion efficiencies. The perovskite crystals maintained their cubic symmetry even at 5% addition of Cu at the Pb site. The effects of slight incorporation of transition metals (*e.g.* Cr^2+^, Co^2+^, Cu^2+^, and Y^3+^) into the HN=CHNH_3_PbI_3_ (FAPbI_3_) perovskite compounds on the electronic structure, chemical shift and optical absorption spectra have been investigated through first-principle calculations.^48,49^ The band gap decreases readily through addition of transition elements because of their influence on the density of states (DOS) through the 3d orbital. Other approaches to improving current density include reports of PbI_2_ addition to CH_3_NH_3_PbI_3−*x*_Cl precursor solution.^50–52^ Recombination between holes and electrons is suppressed by PbI_2_ addition, which improves conversion efficiencies.

The purpose of the present study is to fabricate and characterize alkali metal elements with added Cu CH_3_NH_3_PbI_3−*x*_Cl_*x*_ photovoltaic devices. We performed first-principles calculations to investigate the electronic structures. Elements with ionic radii smaller than MA^+^ (2.17 Å) were added to the solution to compensate for the lattice distortion caused by Cu substitution at Pb sites. Thus, we discuss the effects of alkali metal iodides (*i.e.* NaI, KI, RbI, and CsI) and CuBr_2_ addition. We expect that MA can be easily substituted by alkali metal elements in the perovskite crystal, and the alkali metal elements contribute to improved stability of the perovskite photovoltaic devices. The carrier transport mechanism was investigated by analyzing electronic structures and stabilities of the cells.

## Experimental

### Device fabrication

**Fig. 1:**
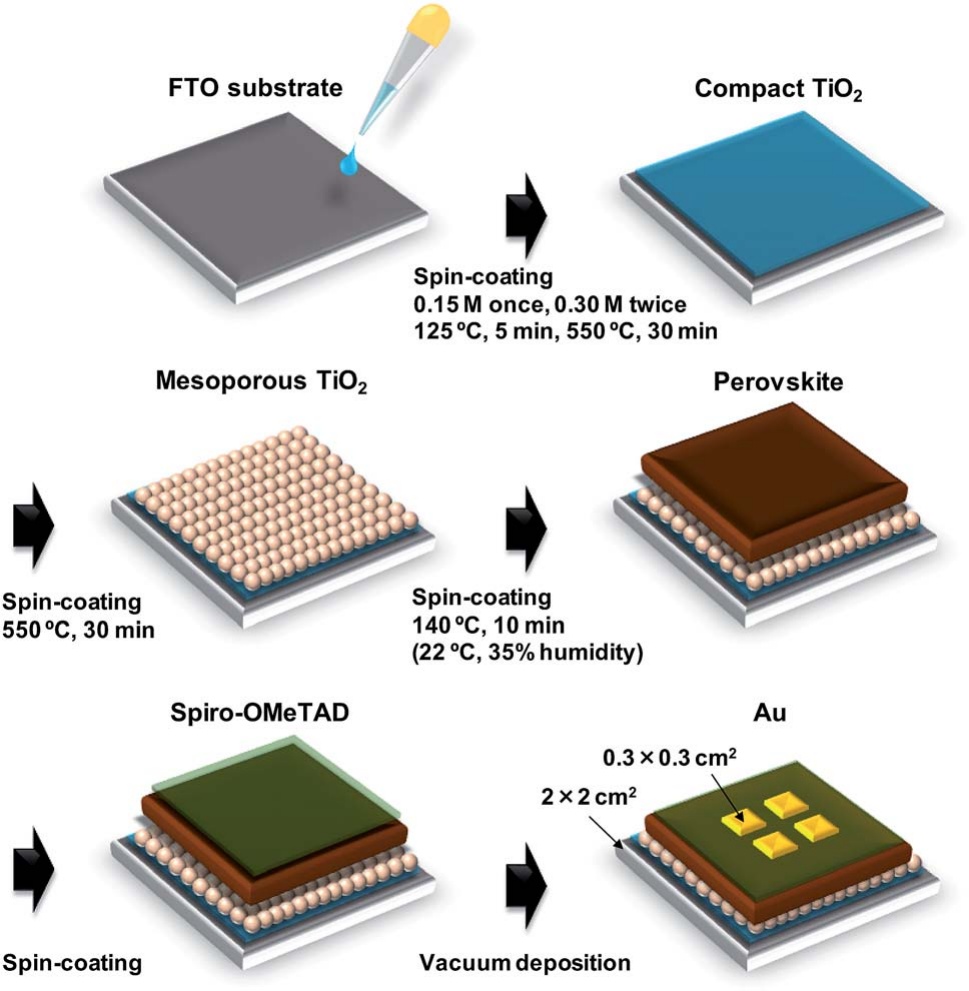
Schematic illustration of fabrication process of the present photovoltaic devices.

A schematic illustration of the fabrication process of the perovskite photovoltaic cells is shown in Fig. 1. F-Doped tin oxide (FTO) substrates were cleaned in an ultrasonic bath with acetone and methanol and then dried under nitrogen gas. The TiO_2_ (0.15 M and 0.30 M) precursor solutions were prepared from titanium diisopropoxide bis(acetyl acetonate) (Sigma-Aldrich, Tokyo, Japan, 0.055 and 0.11 mL) with 1-butanol (1 mL). The 0.15 M TiO_2_ precursor solution was spin-coated on the FTO substrate at 3000 rpm for 30 s and the coated substrate was then annealed at 125 °C for 5 min. Then, the 0.30 M TiO_*x*_ precursor solution was spin-coated on the TiO_2_ layer at 3000 rpm for 30 s and the resulting substrate was annealed at 125 °C for 5 min. The process for forming the 0.30 M precursor layer was performed twice. Then, the FTO substrate was sintered at 550 °C for 30 min to form a compact TiO_2_ layer.^53,54^ To form the mesoporous TiO_2_ layer, a TiO_2_ paste was prepared from the TiO_2_ powder (Aerosil, Tokyo, Japan, P-25) with poly(ethylene glycol) (Nacalai Tesque, Kyoto, Japan, PEG #20000) in ultrapure water. The solution was mixed with acetylacetone (Wako Pure Chemical Industries, Osaka, Japan, 10 μL) and surfactant (Sigma-Aldrich, Triton X-100, 5 μL) for 30 min and was then allowed to stand for 24 h to remove bubbles from the solution. Then, the TiO_2_ paste was spin-coated on the compact TiO_2_ layer at 5000 rpm for 30 s. The resulting cell was heated at 125 °C for 5 min and then annealed at 550 °C for 30 min to form the mesoporous TiO_2_ layer.^55,56^

To prepare the perovskite compounds, mixed solutions of CH_3_NH_3_I (2.4 M, Showa Chemical, Tokyo, Japan), PbCl_2_ (0.8 M, Sigma-Aldrich) and PbI_2_ (0.08 M, Sigma-Aldrich) in DMF (Sigma-Aldrich, 500 mL) were prepared for the standard cell. Details of the perovskite solutions with CuBr_2_ and alkali metal elements are listed in Table S1.† These perovskite solutions were then introduced into the TiO_2_ mesopores by spin coating at 2000 rpm for 60 s, followed by annealing at 140 °C for 10 min in ambient air.^46^

Then, a hole-transport layer was prepared by spin coating; a solution of spiro-OMeTAD (Wako Pure Chemical Industries, 50 mg) in chlorobenzene (Wako Pure Chemical Industries, 0.5 mL) was mixed with a solution of lithium bis(trifluoromethylsulfonyl)imide (Li-TFSI; Tokyo Chemical Industry, Tokyo, Japan, 260 mg) in acetonitrile (Nacalai Tesque, 0.5 mL) for 24 h. The former solution with 4-*tert*-butylpyridine (Sigma-Aldrich, 14.4 μL) was mixed with the Li-TFSI solution (8.8 μL) for 30 min at 70 °C. Then, the spiro-OMeTAD solution was spin-coated on the perovskite layer at 4000 rpm for 30 s. All procedures were performed in air.

Finally, gold (Au) electrodes were evaporated as top electrodes using a metal mask for the patterning. Layered structures of the prepared photovoltaic cells are denoted as FTO/TiO_2_/perovskite/spiro-OMeTAD/Au. The prepared perovskite photovoltaic devices were stored at 22 °C and ∼30% humidity.

### Calculations characterization

*J*–*V* characteristics of the photovoltaic cells were measured under illumination at 100 mW cm^−2^, with the use of an AM 1.5 solar simulator (San-ei Electric, XES-301S). *J*–*V* measurements were performed using a source measurement unit (Keysight, B2901A Precision SMU). The scan rate and sampling time were ∼0.08 V s^−1^ and 1 ms, respectively. Four cells were tested for each cell composition and the reported values are the averages of these four measurements (*η*_ave_). The solar cells were illuminated through the sides of the FTO substrates and the illuminated area was 0.0784 cm^2^. EQEs (Enli Technology, QE-R) of the cells were also measured using a source meter (Keithley Tektronix, 2450). The microstructures of the cells were investigated by X-ray diffraction (XRD, Bruker, D2 PHASER) and scanning electron microscopy (SEM, JEOL, JSM-6010PLUS/LA) equipped with energy dispersive X-ray spectroscopy (EDS).

### Calculations

The electronic structures of the perovskite crystals were calculated for the single-point with the experimental parameters obtained from XRD, through *ab initio* quantum calculations based on the unrestricted Hartree–Fock (HF) method. We used density functional theory (DFT) and the Perdew–Burke–Ernzerhof (PBE)-based hybrid function and unrestricted B3LYP (UB3LYP) with LANL2MB as the basis set (Gaussian 09). The metal-incorporated MAPbI_3_ cubic structures were treated as a cluster model with supercells of 2 × 2 × 2 fixed to have a positive charge of +8. A lattice constant of 6.391 Å was used for the perovskite compounds with a cubic crystal system.^55,57^ The numbers of quantum spins in the metal (M)-incorporated MAPbI_3_ and MAPbI_3_ crystals were assumed to be doublet (*S* = 1/2) states at M = Cu^2+^ and a singlet (*S* = 0) state at M = Pb^2+^, respectively. As an isolated dilution system, the mole ratio of the transition metal to Pb metal was adjusted to be 1 : 26. The concentration of the metal atom was maintained at less than 5% so as not to break crystal symmetry by suppression of strong exchange interactions in the perovskite crystal. Alkali metal elements (*i.e.*, Na^+^, K, Rb^+^, and Cs^+^) were substituted for MA^+^ sites at contents of less than 12%. We calculated the total and partial DOS (TDOS and pDOS), the occupancy of the 3d orbital on the transition metal, 6s, 5p, and 6p orbitals of the I and Pb atoms around the highest occupied molecular orbital (HOMO), and lowest unoccupied molecular orbital (LUMO), and the HOMO–LUMO energy gap (*E*_g_). The vibration modes in infrared spectroscopy (IR) spectra were also calculated by DFT using the frequency mode. The Gibbs energy (*G*) and enthalpy (*H*) were obtained by the IR calculation, and the entropy was calculated by using *G* = *H* − *TS*, where *T* is 298 K.

## Results and discussion

**Fig. 2:**
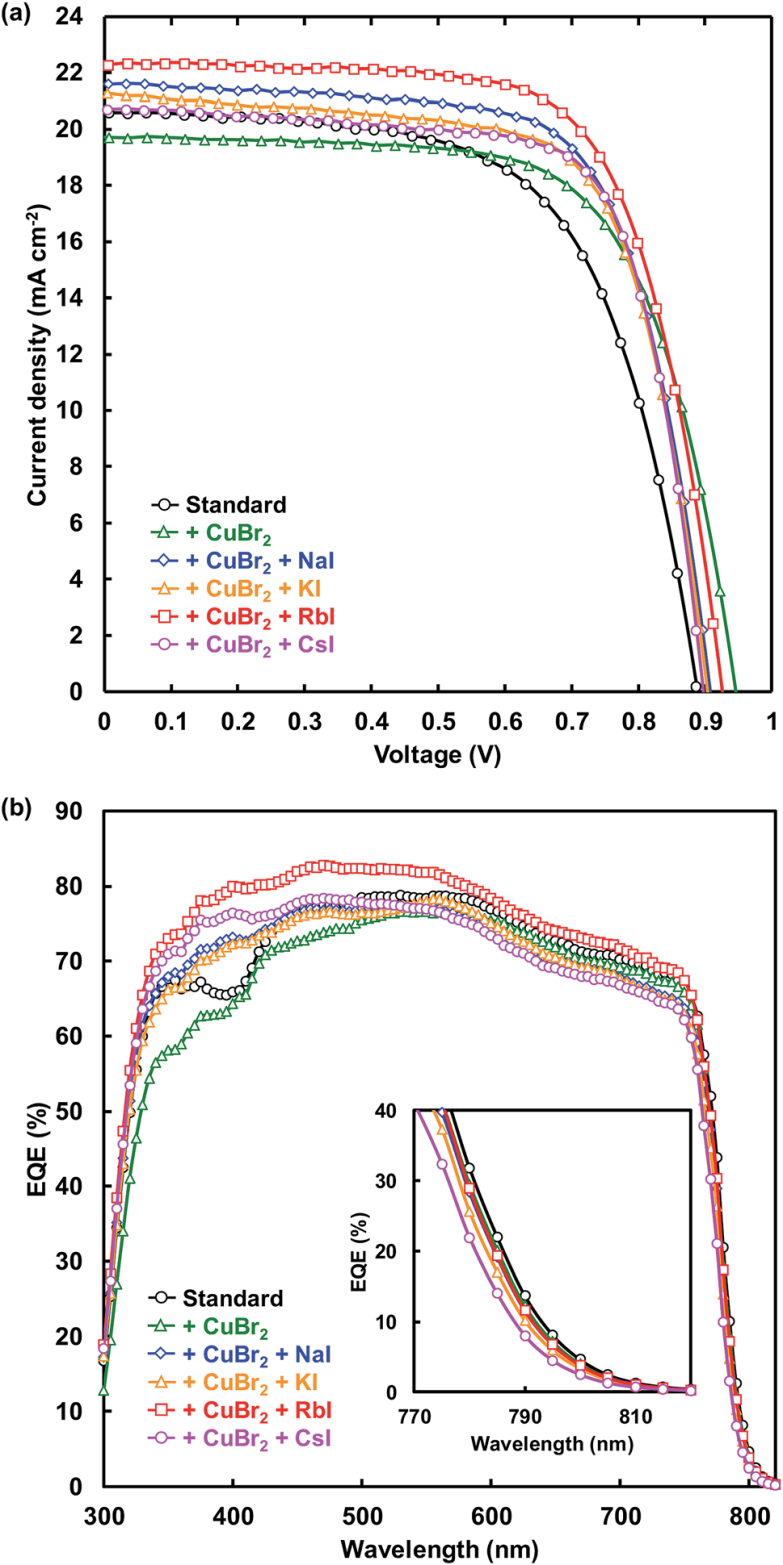
(a) *J*–*V* characteristics and (b) EQE of the present perovskite photovoltaic devices.

*J*–*V* characteristic under illumination recorded in the reverse scan and EQE spectra of FTO/TiO_2_/perovskite/spiro-OMeTAD/Au photovoltaic devices are shown in Fig. 2(a) and (b), respectively. Measured photovoltaic parameters of the present perovskite photovoltaic devices are summarized in Table 1. The standard cell provided a short-circuit current density (*J*_SC_) of 20.6 mA cm^−2^, an open-circuit voltage (*V*_OC_) of 0.888 V, a fill factor (FF) of 0.628, and a conversion efficiency (*η*) of 11.5%. The *V*_OC_ and *η* respectively increased from 0.888 V and 11.5% to 0.946 V and 12.6% through addition of CuBr_2_. Although the series resistance (*R*_S_) increased from 5.02 to 5.87 Ω cm^2^ through CuBr_2_ addition, *R*_S_ decreased to 4.77 Ω cm^2^ by simultaneous addition of CuBr_2_ and RbI. In addition, the shunt resistance (*R*_Sh_) was increased from 2330 to 4664 Ω cm^2^, and the leakage current was decreased by the CuBr_2_ and RbI addition. As a result, *η* increased from 11.5% to 14.2% for the simultaneous addition of CuBr_2_ and RbI. The FF also increased from 0.628 to 0.719 and the recombination between electrons and holes was suppressed by CuBr_2_ and CsI addition.

**Table 1:** Measured photovoltaic parameters of the present perovskite photovoltaic devices

Cells	*J*_SC_ (mA cm^−2^)	*V*_OC_ (V)	FF	*η* (%)	*η*_ave_ (%)	*R*_S_ (Ω cm^2^)	*R*_Sh_ (Ω cm^2^)
Standard	20.6	0.888	0.628	11.5	10.8	5.02	2330
+CuBr_2_	19.7	0.946	0.674	12.6	12.3	5.87	2205
+CuBr_2_ + NaI	21.6	0.907	0.691	13.6	12.8	4.64	1068
+CuBr_2_ + KI	21.3	0.903	0.688	13.2	12.4	4.60	618
+CuBr_2_ + RbI	22.3	0.925	0.690	14.2	13.8	4.77	4664
+CuBr_2_ + CsI	20.7	0.897	0.719	13.3	13.1	4.05	1131

EQE values were increased by alkali metal elements addition in the range of 300–500 nm. The highest EQE of the device increased from 78.8% to 82.8% by RbI addition, indicating that the current density of the cells was increased by the RbI addition. The band gaps of the present perovskite photovoltaic devices estimated from EQE spectra are summarized in Table 2. The band gap was increased by addition of CuBr_2_ and alkali metal elements. In particular, the band gap increased from 1.583 to 1.594 eV by CsI addition.

**Table 2:** Band gaps of the present perovskite photovoltaic devices estimated by EQE

Cells	Band gap (eV)
Standard	1.583
+CuBr_2_	1.585
+CuBr_2_ + NaI	1.587
+CuBr_2_ + KI	1.590
+CuBr_2_ + RbI	1.588
+CuBr_2_ + CsI	1.594

**Fig. 3:**
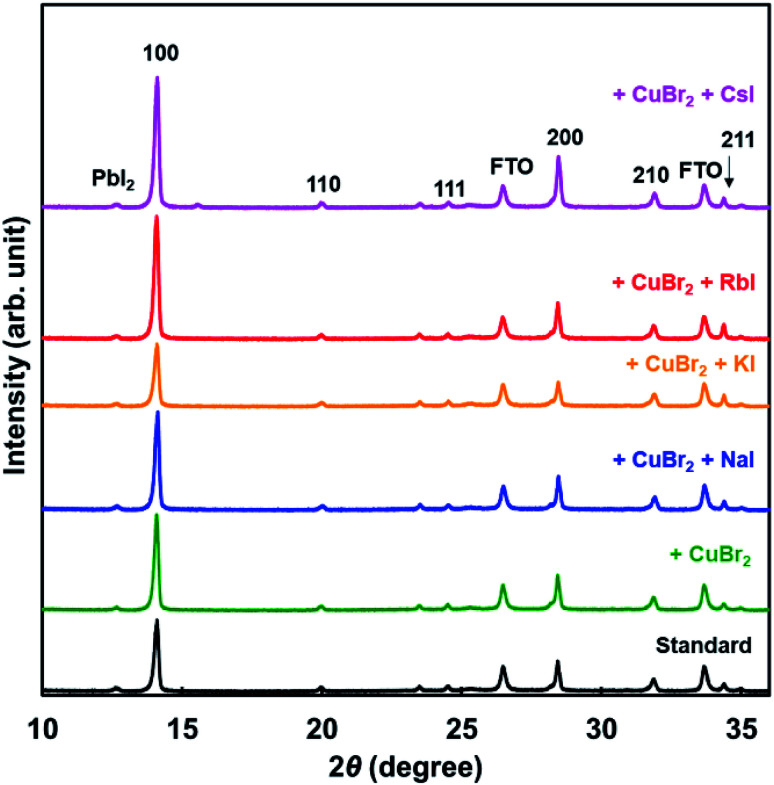
XRD patterns of the present perovskite photovoltaic devices.

XRD patterns of the present photovoltaic devices are shown in Fig. 3. The crystal structure of the standard cell was a cubic system, and the cells added with CuBr_2_ and alkali metal elements also had a cubic system. Diffraction intensities of the 210 peak were the same in all devices. However, diffraction intensities of 100 increased through addition of NaI, RbI, and CsI. Hence, the 100 planes of the perovskite grains were preferentially oriented parallel to the FTO substrate.^46^ Measured XRD parameters of the present perovskite photovoltaic devices are summarized in Table 3. The lattice constant was slightly increased by addition of CuBr_2_, compared with the standard constant. Although the lattice constant might be expected to decrease owing to the smaller ionic radius of Cu and Br,^58^ the increase of the lattice distance suggested that lattice distortion was caused by CuBr_2_ addition. A tolerance factor of the hypothetical perovskite MACuCl_3_ was calculated to be 1.004, which is closer to 1 compared with that of MAPbI_3_.^59^ A remarkable difference of the lattice constant was not observed because of the very small amount of iodide addition. Analysis of the 100 diffraction peaks of the perovskite crystals suggested the crystallite sizes to be approximately 600 Å.

**Table 3:** Measured XRD parameters of the present perovskite photovoltaic devices

Cells	Lattice constant (Å)	Crystallite size (Å)
Standard	6.279	619
+CuBr_2_	6.282	617
+CuBr_2_ + NaI	6.274	630
+CuBr_2_ + KI	6.279	577
+CuBr_2_ + RbI	6.280	586
+CuBr_2_ + CsI	6.275	604

**Fig. 4:**
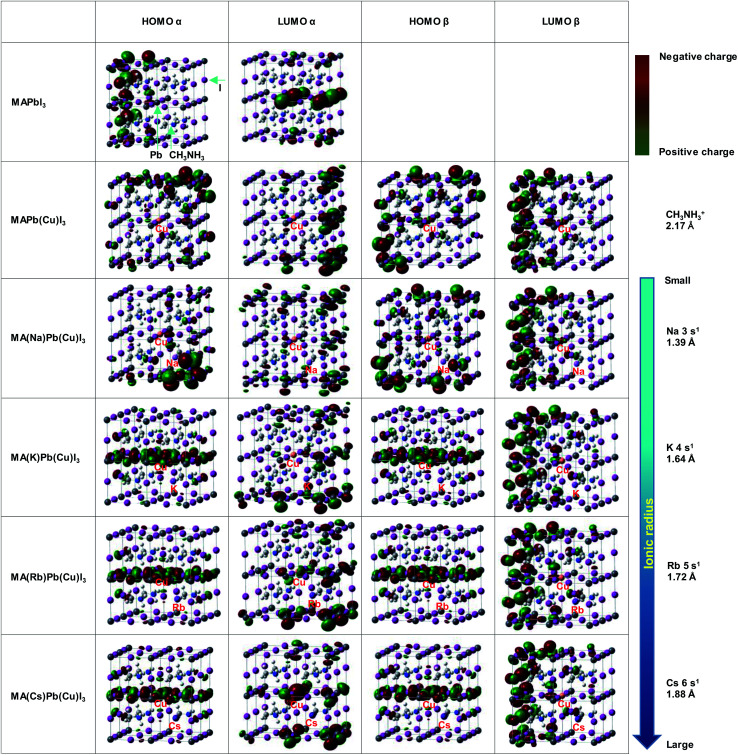
Electronic structures at HOMO and LUMO of the present perovskite structures.

Electronic structures at HOMO and LUMO of the present perovskite crystals were calculated, as shown in Fig. 4. As for the electronic structures, the red and the green parts indicate negative and positive charges, respectively. The electronic charges became broadly distributed by replacing Pb with Cu. These results indicate that the charge carriers were generated more efficiently by the Cu substitution, as shown in Fig. 2.

In the case of Na substituted MA(Na)Pb(Cu)I_3_, some differences were observed; however, the distribution of the electric charge was almost the same as that of MAPb(Cu)I_3_. Because of the small ionic radius of Na, the interaction between Cu and Na is weak, resulting in minimal change of the electric charge distribution. Conversely, for MA(K)Pb(Cu)I_3_, MA(Rb)Pb(Cu)I_3_, and MA(Cs)Pb(Cu)I_3_, we observed a change of the specific electric charge distribution of the HOMO level, owing to interactions of these alkali metal elements with Cu. The linear electric charge distribution would decrease the carrier transport resistance and increase the carrier diffusion length. Thus, the special electric charge distribution formed by CuBr_2_ with NaI, KI, RbI and CsI contributes to the increase of *J*_SC_.

**Fig. 5:**
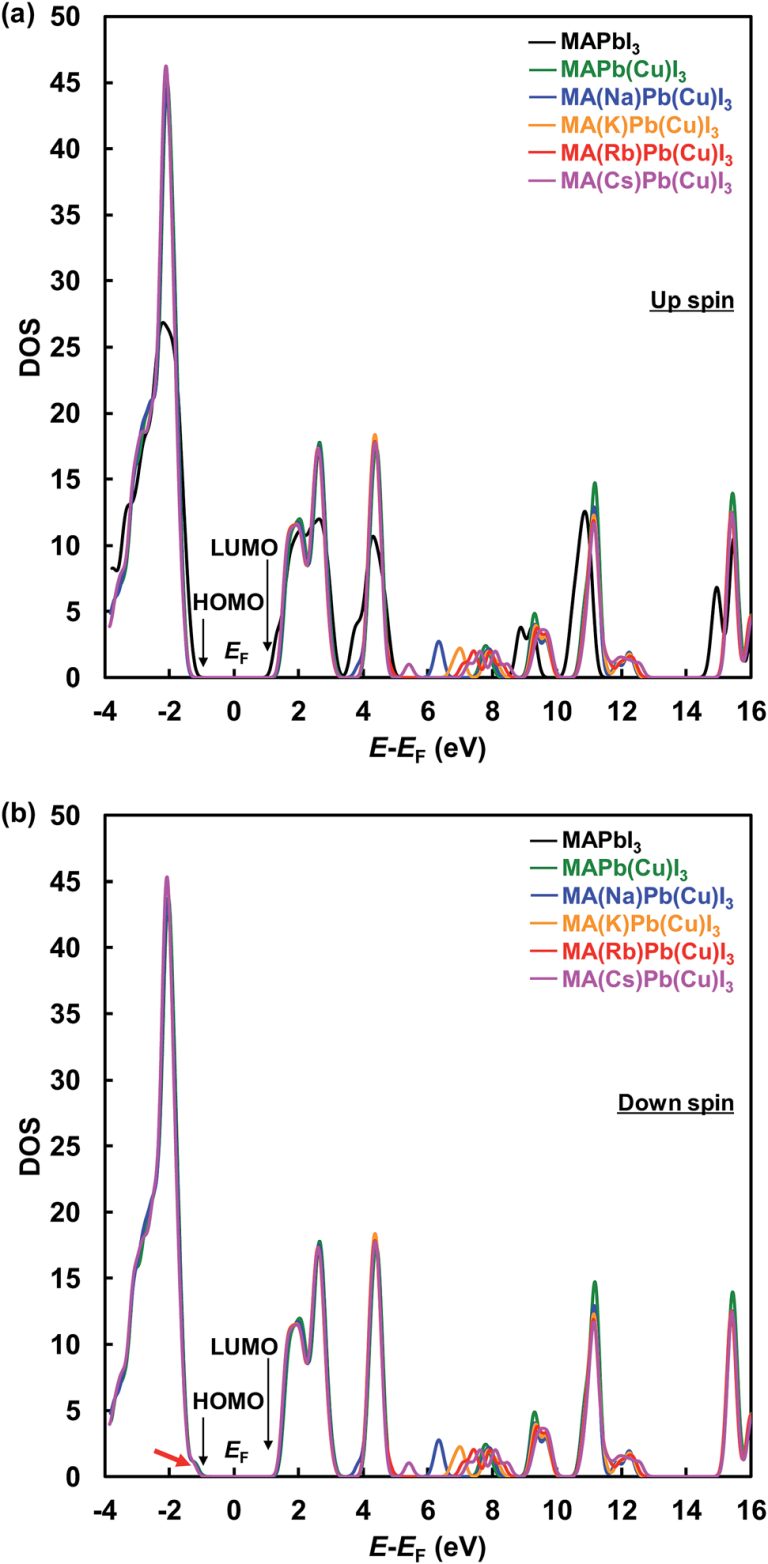
Total density of states with (a) up-α spin and (b) down-β spin of the present perovskite structures.

Total density of states with up-α spin and down-β spin of the present perovskite structures are shown in Fig. 5(a) and (b), respectively. The electronic structure of CH_3_NH_3_PbI_3_ mainly derives from the electronic state of the PbI_6_ octahedron unit, and the level of the energy band is comprised of Pb 6s, 6p and I 5p orbits. The energy level at the HOMO is a σ anti-binding state between Pb 6s and I 5p orbits, and the LUMO is a mixed σ or π anti-binding state between Pb 6p and I 5s or 5p orbits. Therefore, substitution of other elements at Pb sites influences the HOMO and LUMO levels. The DOS at the HOMO was greatly increased by Cu substitution but not influenced by the alkali metal substitution. Hence, the 3d orbital of Cu influences the energy level at HOMO and contributes to an increase in carrier generation. The characteristic increased DOS due to alkali metal elements is observed in the range of 5–10 eV, which would contribute the increased generation of charge carriers by light absorption at short wavelengths. Thus, the EQE improved in the range of 300–500 nm.

These results indicate that alkali metal elements make a contribution to the improved carrier transport properties. As shown by the red arrow in Fig. 5(b), the energy level at the HOMO slightly approached the Fermi level and became sharper, which contributed to the increased hole mobility. Calculated energy levels at the HOMO and LUMO, and the energy gaps (*E*_g_) with α spin of the present perovskite structures are summarized in Table 4. The calculated *E*_g_ values are larger than those of experimental data in Table 2, and such differences have been previously reported for the cluster-model calculations.^60^ The *E*_g_ increased from 2.70 to 3.23 eV by Cu substitution, which increased the *V*_OC_. In addition, the energy gaps decreased in the order Cs^+^ > Rb^+^ > K^+^ > Na^+^. The hole transport properties between the perovskite and spiro-OMeTAD layers benefited from the shift of the DOS at the HOMO to the Fermi level and the decrease in the energy barrier. Therefore, the increase of the *J*_SC_ and *V*_OC_ are attributed to interactions of the Cu and alkali metals, as observed from their electronic structures and DOS.

**Table 4:** Energy levels of the HOMO and LUMO, and energy gap (*E*_g_) with α spin for the present perovskite structures

Perovskite	LUMO (eV)	HOMO (eV)	*E*_g_ (eV)	*E*_F_ (eV)
MAPbI_3_	−14.9	−17.6	2.70	−16.2
MAPb(Cu)I_3_	−14.6	−17.8	3.23	−16.2
MA(Na)Pb(Cu)I_3_	−14.6	−17.8	3.22	−16.2
MA(K)Pb(Cu)I_3_	−14.6	−17.7	3.17	−16.2
MA(Rb)Pb(Cu)I_3_	−14.6	−17.7	3.16	−16.2
MA(Cs)Pb(Cu)I_3_	−14.6	−17.7	3.15	−16.2

**Fig. 6:**
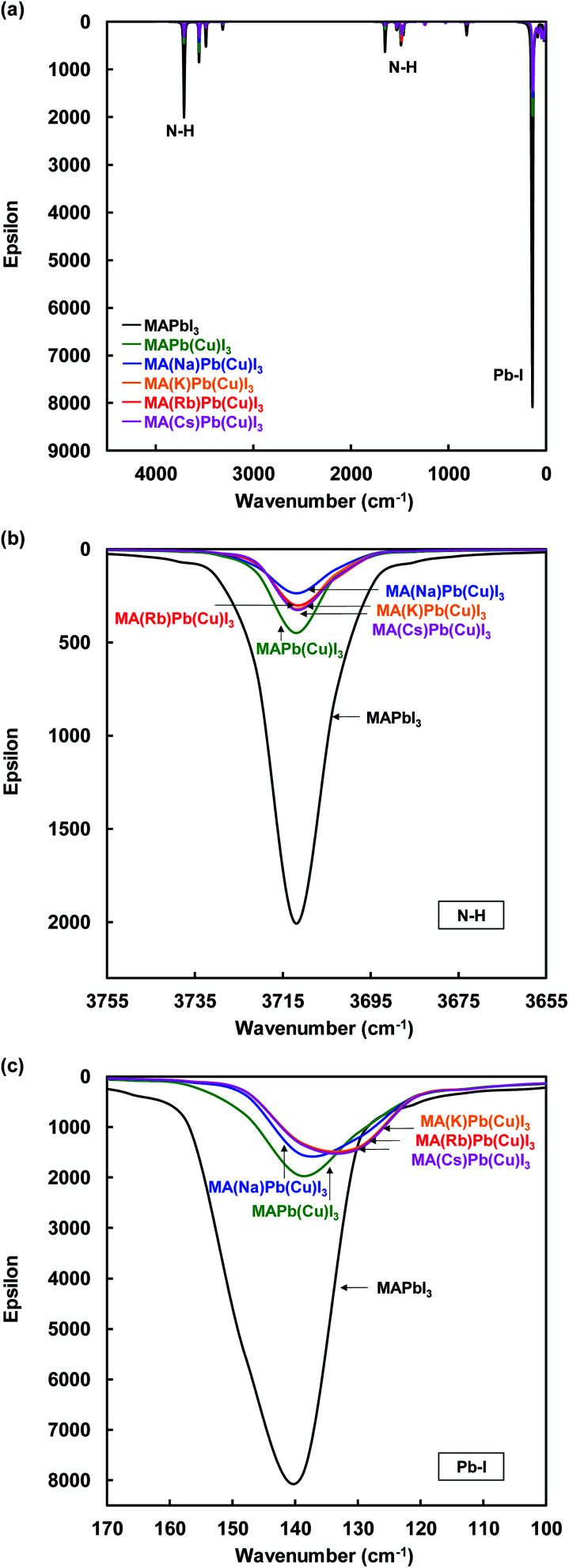
IR spectra of (a) total view, (b) N–H, and (c) Pb–I stretching vibration of the present perovskite structures.

IR spectra of the present perovskite structures are shown in Fig. 6. Both stretching vibrations of N–H and Pb–I were suppressed by Cu substitution, which caused diffusion of the carrier transport resistance and contributed to the increase of the *J*_SC_. In addition, the suppression of the N–H stretching vibration depended on the ionic radius of the alkali metal elements.

Calculated thermodynamic parameters (*G*: Gibbs energy, *H*: enthalpy and *S*: entropy) of the present perovskite structure are summarized in Table 5. As a result of the calculation, the *G*, *H*, and *S* values of the MAPbI_3_ perovskite structure were 946, 2326, and 4.63 kJ K^−1^ mol^−1^, respectively. The value of *G* slightly increased from 946 to 958 kJ mol^−1^ through Cu substitution, which increased the lattice constant of the perovskite with added CuBr_2_, owing to the Jahn–Teller effect of Cu d electrons.^59,61^ The Jahn–Teller effect is often observed for octahedron complexes with transition metals, where the repulsion between d orbital is relaxed by distortion of the arrangement of PbI_6_ octahedra in the perovskite structure. Thus, addition of a large amount of transition metal distorts the perovskite structure. We expected that the lattice distortion of the perovskite crystal including Cu was relaxed by Na^+^, K^+^, Rb^+^, and Cs^+^ substitution.

**Table 5:** Calculated thermodynamic parameters of the present perovskite structures (*G*: Gibbs energy, *H*: enthalpy and *S*: entropy)

Perovskite	*G* (kJ mol^−1^)	*H* (kJ mol^−1^)	*S* (kJ K^−1^ mol^−1^)
MAPbI_3_	946	2326	4.63
MAPb(Cu)I_3_	958	2310	4.54
MA(Na)Pb(Cu)I_3_	756	2074	4.42
MA(K)Pb(Cu)I_3_	746	2076	4.46
MA(Rb)Pb(Cu)I_3_	743	2078	4.48
MA(Cs)Pb(Cu)I_3_	729	2084	4.55

The value of *G* gradually decreased to 729 kJ mol^−1^, and more stable perovskite structures were formed by alkali metal substitution in order of: Cs^+^ > Rb^+^ > K^+^ > Na^+^ substitution. Therefore, the substitution with alkali metal elements effectively stabilized the perovskite solar cells. However, further improvements to the solvent system are necessary because Cs compounds are poorly soluble in DMF.

**Fig. 7:**
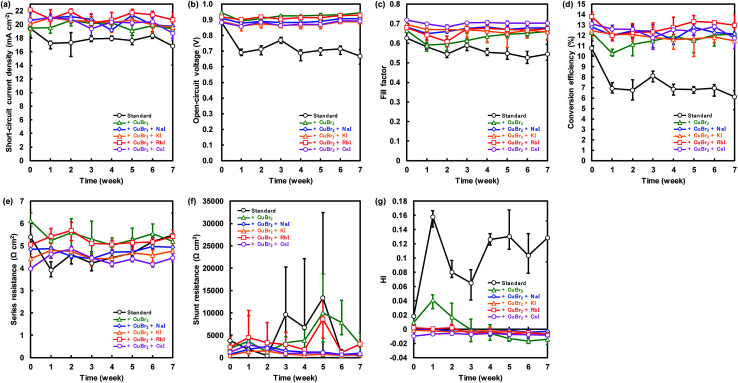
Changes in (a) short-circuit current density (*J*_SC_), (b) open-circuit voltage (*V*_OC_), (c) fill factor (FF), (d) conversion efficiency (*η*), (e) series resistance (*R*_S_), (f) shunt resistance (*R*_Sh_), and (g) hysteresis index (HI) for the present perovskite photovoltaic devices.

Changes of the photovoltaic parameters over 7 weeks are shown in Fig. 7(a)–(f). After *J*–*V* measurements, all devices were stored in thermostatic and humidistatic storage. Hysteresis index (HI) values are also shown in Fig. 7(g). The HI was derived from the data in Fig. 7(a)–(c) according to the following equation:^62^5
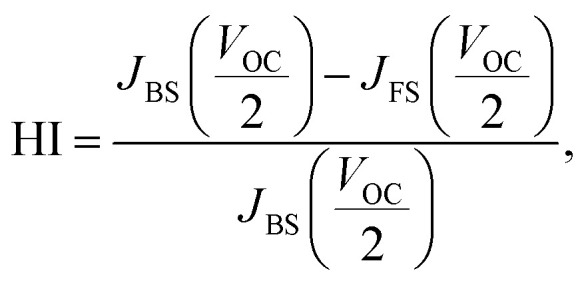
where *J*_BS_ (*V*_OC_/2) is the photocurrent at half *V*_OC_ for the reverse scan and *J*_FS_ (*V*_OC_/2) is the photocurrent at half *V*_OC_ for the forward scan. An HI of 0 corresponds to a cell without hysteresis, whereas an HI of 1 represents the case where the hysteresis is as high as the magnitude of the photocurrent. The increase in HI of the standard device is caused by lattice defects of the perovskite films. The remarkable decrease in photovoltaic performance of the device with added PbI_2_ is compared with the result of a previous study.^52^ The *η* of the device with added CuBr_2_ remained almost constant for 7 weeks. However, we confirmed that the HI values increased. The HI values of the devices with added CuBr_2_ and alkali metal remained constant for 7 weeks. Thus, the decomposition of perovskite grains was suppressed by CuBr_2_ and alkali metal substitutions.

The *J*_SC_, *V*_OC_, and *η* of the standard device were 17.4 mA cm^−2^, 0.711 V and 6.7%, respectively, after 7 weeks. Conversely the device with added CuBr_2_ and RbI provided the best *J*_SC_, *V*_OC_, and *η* of 21.8 mA cm^−2^, 0.937 V and 14.0%, respectively after 7 weeks, which resulted in improved stability of the perovskite device. We considered that the improved stability is associated with lattice defects. MA can be substituted easily by alkali metal elements in the perovskite crystal, and the alkali metal elements can contribute to the stability of perovskite photovoltaic devices. Moreover, the device with added CuBr_2_ and CsI maintained the highest FF of more than 0.7 after 7 weeks. These results are consistent with our first-principle calculations.

## Conclusions

We investigated the effects of alkali metal (Na^+^, K^+^, Rb^+^, and Cs^+^) additives on CH_3_NH_3_PbI_3−*x*_Cl_*x*_ photovoltaic devices, which also contained Cu, by device performance experiments and first-principles calculation. Our DOS calculations indicated that the *E*_g_ and DOS of the HOMO level were increased by Cu substitution at Pb sites. The DOS of alkali metal elements was effective for the carrier generation as observed in the EQE in range of 300–500 nm. An IR calculation showed that the Pb–I stretching vibration was suppressed. Although the value of *G* was increased by Cu substitution, *G* decreased through addition of alkali metal elements. From these results, we attribute the decrease in series resistance and increased stability to the simultaneous addition of Cu and Rb. The *J*_SC_ and *V*_OC_ increased to 22.3 mA cm^−2^ and 0.925 V from 20.6 mA cm^−2^ and 0.888 V, respectively, through the addition of CuBr_2_ and RbI to the CH_3_NH_3_PbI_3−*x*_Cl_*x*_ perovskite precursor solution; conversion efficiency also increased from 11.5% to 14.2%. Furthermore, the formation of HI was decreased, and the stability was improved by addition of CuBr_2_ and RbI. These experimental results agreed well with our calculations, confirming the usefulness of interactions between Cu and alkali metal elements. The present work will provide a possible guideline to improve both carrier transport and stability of the perovskite solar cells by simultaneous addition of CuBr_2_ and alkali metals.

## Conflicts of interest

There are no conflicts to declare.

## Supplementary Material

RA-009-C9RA03068A-s001
