# Supplementary material for: Additive effects of alkali metals on Cu-modified CH3NH3PbI3−δClδ photovoltaic devices
Source: RSC Adv. 2019 Aug 5;9(42):24231–40. doi: 10.1039/c9ra03068a (PMC9069633; doi:10.1039/c9ra03068a)
Supplement: RA-009-C9RA03068A-s001 [file RA-009-C9RA03068A-s001.pdf]

Electronic Supplementary Material (ESI) for RSC Advances.
This journal is © The Royal Society of Chemistry 2019

Supplementary information

Additive effects of alkali metals on Cu-modified $CH_3NH_3PbI_{3-\delta}Cl_\delta$ photovoltaic devices

Naoki Ueoka, Takeo Oku, and Atsushi Suzuki

Table S1. Preparation composition of the present perovskite crystals.

Cells	CH_3NH_3I	$PbICl_2$	PbI_2	$CuBr_2$	NaI	KI	RbI	CsI
Standard	2.4 M	0.8 M	0.08 M					
+ $CuBr_2$	2.4 M	0.792 M	0.08 M	0.008 M				
+ $CuBr_2$ + NaI	2.376 M	0.792 M	0.08 M	0.008 M	0.008 M			
+ $CuBr_2$ + KI	2.376 M	0.792 M	0.08 M	0.008 M		0.008 M		
+ $CuBr_2$ + RbI	2.376 M	0.792 M	0.08 M	0.008 M			0.008 M	
+ $CuBr_2$ + CsI	2.376 M	0.792 M	0.08 M	0.008 M				0.008 M

Table S2. Energy levels of the HOMO and LUMO, and energy gap (E_g) of the perovskite structures without Cu, calculated by first-principles calculation.

Perovskite	LUMO (eV)	HOMO (eV)	E_g (eV)	E_F (eV)
$MAPbI_3$	-14.9	-17.6	2.70	-16.2
$MA(Na)PbI_3$	-14.9	-17.6	2.71	-16.2
$MA(K)PbI_3$	-14.9	-17.6	2.71	-16.2
$MA(Rb)PbI_3$	-14.9	-17.6	2.71	-16.2
$MA(Cs)PbI_3$	-14.9	-17.6	2.71	-16.2

Table S3. Calculated thermodynamic parameters of the perovskite structures without Cu. (*G*: Gibbs energy, *H*: enthalpy and *S*: entropy.)

Perovskite	G (kJ mol^{-1})	H (kJ mol^{-1})	S (kJ K^{-1} mol^{-1})
$MAPbI_3$	946	2326	4.63
$MA(Na)PbI_3$	728	2092	4.58
$MA(K)PbI_3$	741	2090	4.53
$MA(Rb)PbI_3$	721	2094	4.61
$MA(Vs)PbI_3$	722	2097	4.61

Supplementary information

Table S4. Measured photovoltaic parameters of the present perovskite photovoltaic devices.

	J_{SC} (mA cm^{-2})	V_{OC} (V)	FF	η (%)	η_{ave} (%)	R_S (Ω cm^2)	R_{Sh} (Ω cm^2)
After 1 week							
Standard	17.5	0.713	0.601	7.5	6.9	3.62	3788
+ $CuBr_2$	20.6	0.892	0.580	10.7	10.3	5.12	1344
+ $CuBr_2$ + NaI	21.6	0.882	0.653	12.5	12.0	4.83	2440
+ $CuBr_2$ + KI	21.6	0.878	0.676	12.8	12.1	4.88	2200
+ $CuBr_2$ + RbI	22.1	0.900	0.625	12.5	12.0	5.10	1980
+ $CuBr_2$ + CsI	21.4	0.863	0.701	13.0	12.6	4.44	2540
After 2 weeks							
Standard	18.8	0.734	0.562	7.8	6.8	4.49	674
+ $CuBr_2$	21.1	0.917	0.622	12.0	11.2	5.42	1926
+ $CuBr_2$ + NaI	21.7	0.893	0.671	13.0	12.4	4.44	1810
+ $CuBr_2$ + KI	20.6	0.891	0.681	12.5	12.1	4.97	1485
+ $CuBr_2$ + RbI	22.3	0.925	0.637	13.1	12.3	5.23	7785
+ $CuBr_2$ + CsI	21.0	0.883	0.695	12.9	12.6	4.81	1534
After 3 weeks							
Standard	17.5	0.787	0.622	8.6	8.1	3.89	20222
+ $CuBr_2$	20.6	0.928	0.652	12.5	11.5	4.65	4317
+ $CuBr_2$ + NaI	21.5	0.887	0.681	13.0	12.4	4.34	1803
+ $CuBr_2$ + KI	21.2	0.884	0.685	12.8	11.8	4.58	1230
+ $CuBr_2$ + RbI	21.6	0.910	0.673	13.2	12.4	5.10	2224
+ $CuBr_2$ + CsI	20.2	0.860	0.708	12.3	11.8	4.42	1067
After 4 weeks							
Standard	18.0	0.706	0.573	7.3	6.9	4.34	22089
+ $CuBr_2$	20.2	0.930	0.679	12.7	11.9	4.79	5675
+ $CuBr_2$ + NaI	19.3	0.887	0.691	11.8	11.6	4.66	1361
+ $CuBr_2$ + KI	20.8	0.890	0.691	12.8	11.6	4.55	654
+ $CuBr_2$ + RbI	21.8	0.925	0.682	13.7	12.8	4.77	1814
+ $CuBr_2$ + CsI	20.8	0.879	0.707	13.0	12.6	4.29	891
After 5 weeks							
Standard	17.0	0.722	0.578	7.1	6.8	4.48	18415
+ $CuBr_2$	20.7	0.940	0.679	13.2	11.5	4.71	18738
+ $CuBr_2$ + NaI	21.8	0.894	0.684	13.3	12.8	4.54	1426
+ $CuBr_2$ + KI	20.9	0.887	0.679	12.6	11.7	4.85	1325
+ $CuBr_2$ + RbI	22.0	0.918	0.690	13.9	13.3	4.86	10343
+ $CuBr_2$ + CsI	20.7	0.868	0.713	12.8	12.4	4.28	1017
After 6 weeks							
Standard	17.9	0.726	0.559	7.3	6.9	4.87	1733
+ $CuBr_2$	21.3	0.940	0.662	13.3	12.1	5.32	12829
+ $CuBr_2$ + NaI	20.3	0.912	0.683	12.6	12.3	4.85	691
+ $CuBr_2$ + KI	20.6	0.907	0.689	12.9	11.8	4.45	506
+ $CuBr_2$ + RbI	21.0	0.921	0.690	13.3	13.3	5.07	1426
+ $CuBr_2$ + CsI	21.0	0.892	0.712	13.3	12.8	4.05	723
After 7 weeks							
Standard	17.4	0.711	0.543	6.7	6.1	5.04	812
+ $CuBr_2$	20.2	0.946	0.682	13.0	12.1	4.90	5476
+ $CuBr_2$ + NaI	21.2	0.918	0.678	13.2	12.1	4.71	972
+ $CuBr_2$ + KI	20.3	0.909	0.688	12.7	11.4	4.85	894
+ $CuBr_2$ + RbI	21.7	0.937	0.688	14.0	13.0	4.93	4077
+ $CuBr_2$ + CsI	19.5	0.906	0.699	12.4	11.7	4.50	820

Supplementary information

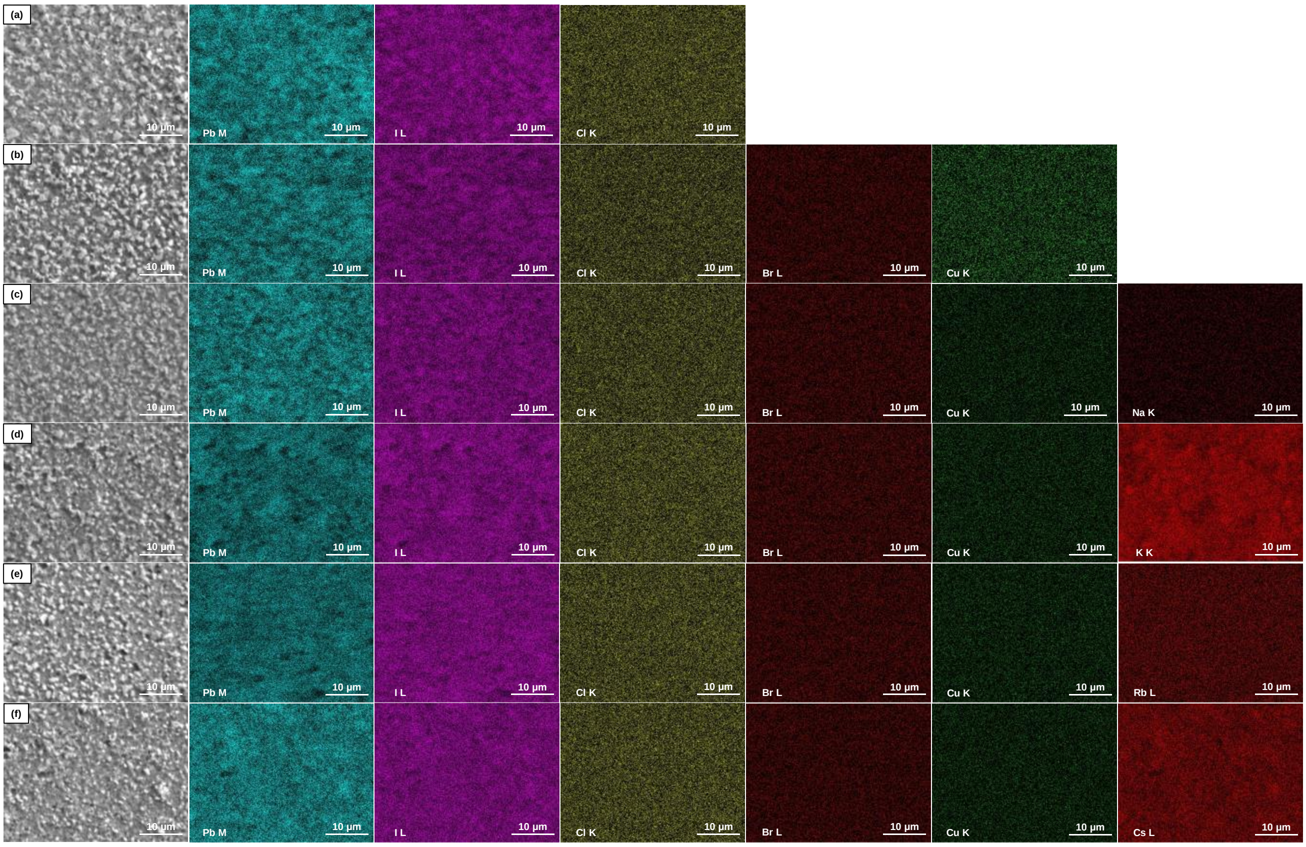

Fig. S1. SEM images and corresponding elemental mappings of the Pb M, I L, Cl K, Br L, Na K, K K, Rb L, and Cs L lines of (a) standard, (b) + $CuBr_2$, (c) + $CuBr_2$ + NaI, (d) + $CuBr_2$ + KI, (e) + $CuBr_2$ + RbI and (f) + $CuBr_2$ + CsI perovskite photovoltaic devices.

Supplementary information

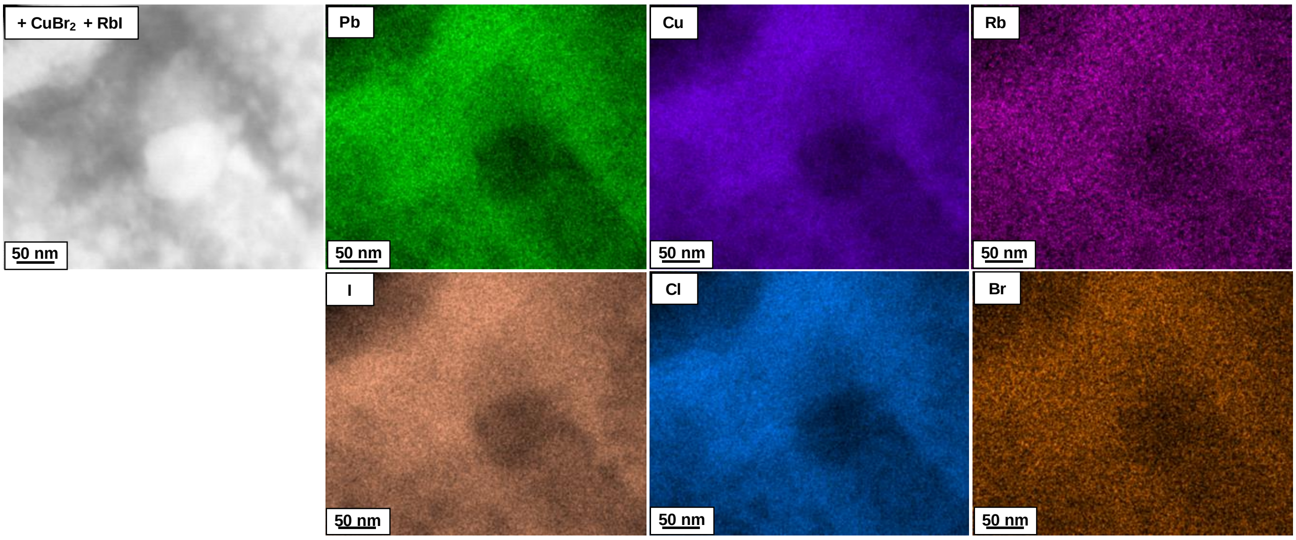

Fig. S2. High-angle annular dark-field scanning transmission electron microscopy image and corresponding elemental mappings of the Pb L, Cu K, Rb K, I K, Cl K, and Br K lines of + $CuBr_2$ + RbI perovskite photovoltaic device.

Supplementary information

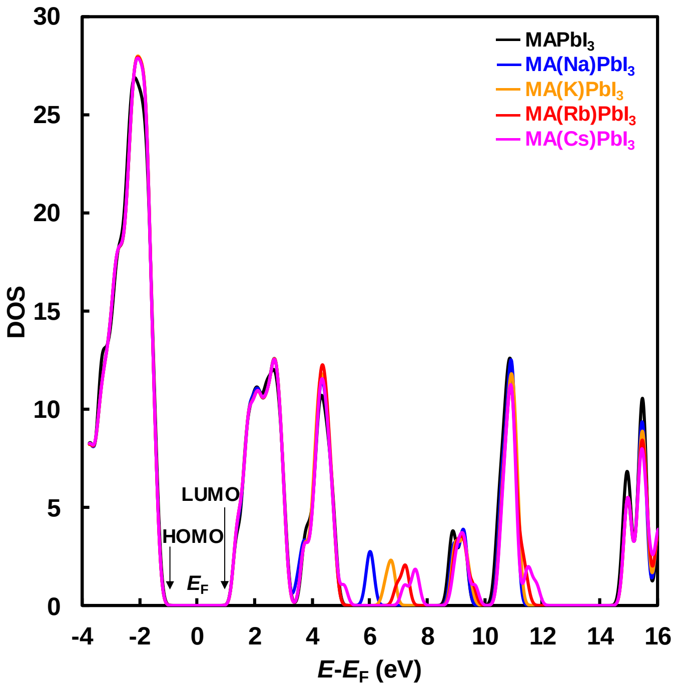

Fig. S3. Total density of states of the present perovskite structures without Cu.

Supplementary information

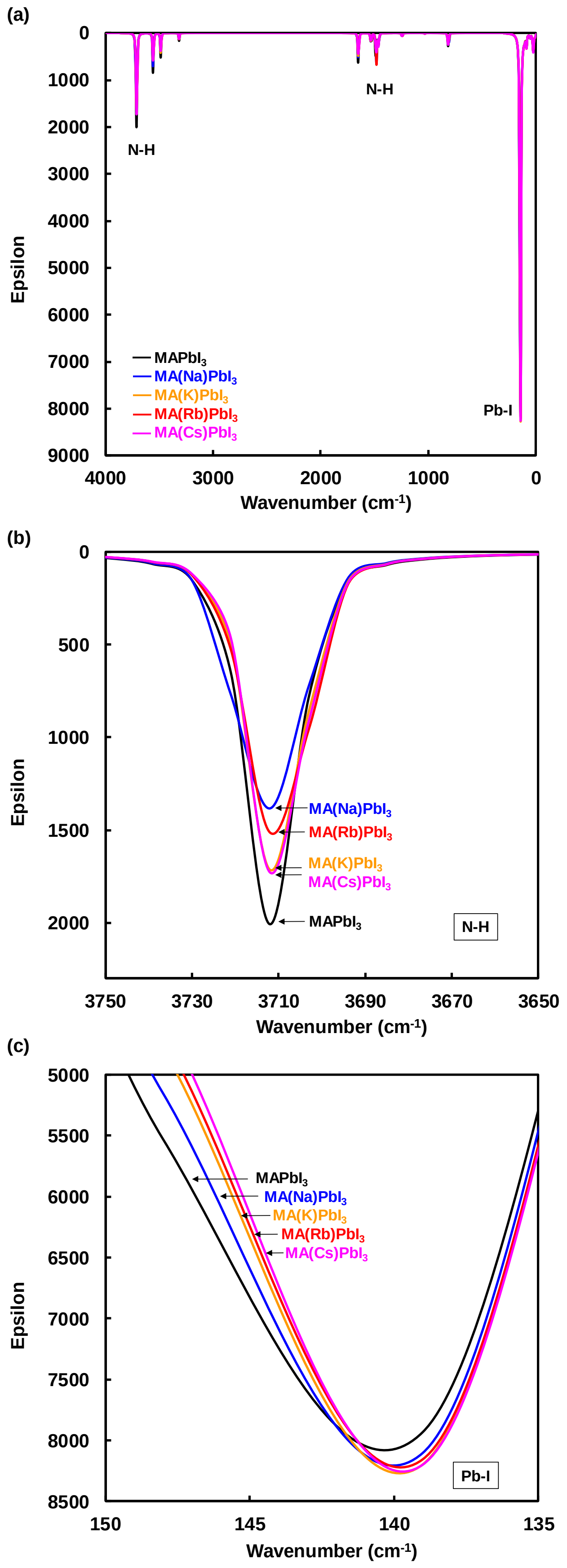

Fig. S4. IR spectra of (a) total view, (b) N-H, and (c) Pb-I stretching vibration of the perovskite structures without Cu.

Supplementary information

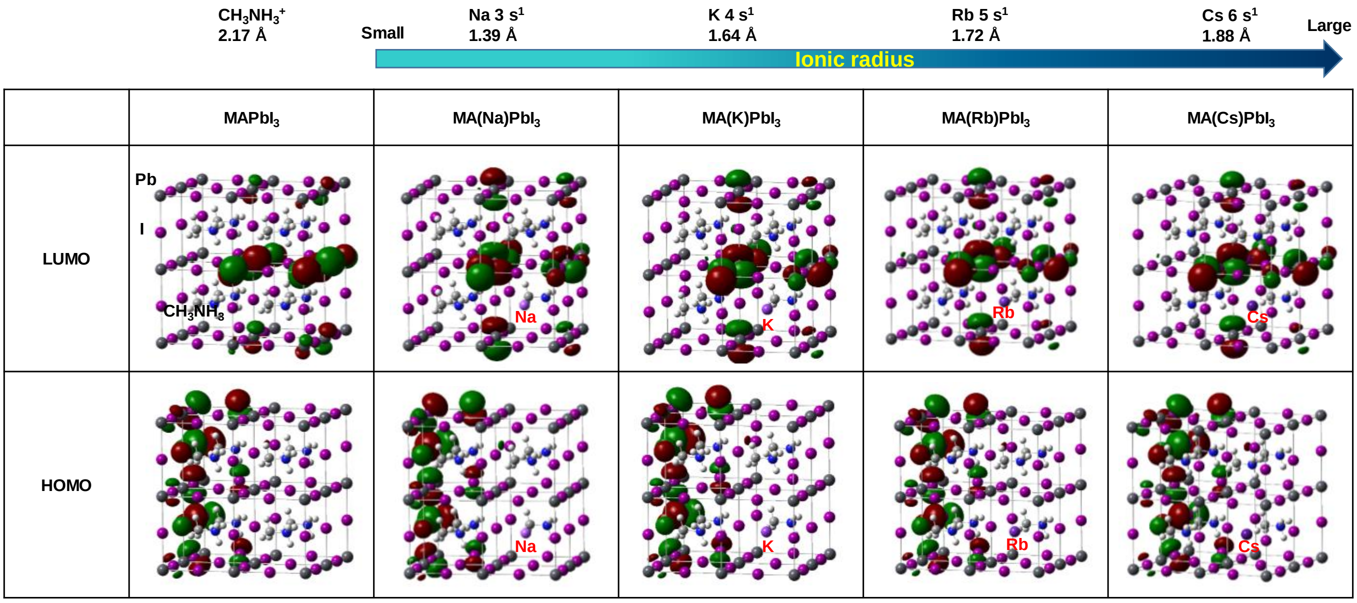

Fig. S5. Electronic structures at HOMO and LUMO of the present perovskite structures without Cu.
